# Repeat *Chlamydia trachomatis* testing among heterosexual STI outpatient clinic visitors in the Netherlands: a longitudinal study

**DOI:** 10.1186/s12879-017-2871-1

**Published:** 2017-12-20

**Authors:** Maartje Visser, Fleur van Aar, Femke D. H. Koedijk, Carolina J. G. Kampman, Janneke C. M. Heijne

**Affiliations:** 1 National Institute for Public Health and the Environment, P.O. Box 1, 3720 BA Bilthoven, the Netherlands; 2 Public Health Service Twente, postbus 1400, 7500 BK Enschede, the Netherlands

**Keywords:** Chlamydia trachomatis, Sexually transmitted infections, Repeat testing, STI clinic, Public health, The Netherlands

## Abstract

**Background:**

Chlamydia infections are common in both men and women, are often asymptomatic and can cause serious complications. Repeat testing in high-risk groups is therefore indicated. In the Netherlands, guidelines on repeat chlamydia testing differ between testing facilities, and knowledge on repeat testing behaviour is limited. Here, we analyse the current repeat testing behaviour of heterosexual STI clinic visitors, and aim to identify groups for which repeat testing advice could be advantageous.

**Methods:**

Longitudinal surveillance data from all Dutch STI outpatient clinics were used, which included all STI clinic consultations carried out among heterosexual men and women between June 2014 and December 2015. Repeat testing was defined as returning to the same STI clinic between 35 days and 12 months after initial consultation. We calculated chlamydia positivity at repeat test stratified by initial test result and time between consultations. Logistic regression analyses were used to identify predictors of repeat testing, and predictors of having a chlamydia positive repeat test.

**Results:**

In total, 140,486 consultations in 75,487 women and 46,286 men were available for analyses. Overall, 15.4% of women and 11.1% of men returned to the STI clinic within the study period. Highest chlamydia positivity at repeat test was seen 3–5 months after initial positive test. Among both women and men, repeat testing was associated with non-Western ethnicity, having had more than two sex partners in the past 6 months, reporting STI symptoms, having a history of STI, and having a chlamydia positive initial test. Among repeat testers, chlamydia positive repeat test was most strongly associated with younger age, followed by a chlamydia positive initial test.

**Conclusions:**

Repeat testing most often resulted in a positive test result among young heterosexuals (<25) and heterosexuals of any age with a chlamydia infection at the initial consultation. Further efforts are needed to determine optimal repeat testing strategies.

**Electronic supplementary material:**

The online version of this article (doi: 10.1186/s12879-017-2871-1) contains supplementary material, which is available to authorized users.

## Background

*Chlamydia trachomatis* (chlamydia) is the most frequently reported bacterial sexually transmitted infection (STI) among heterosexuals [1]. Chlamydia reinfections are also common, with proportions of reinfection of up to 32% in women [2], and 18.3% in men [3]. Chlamydia infections are associated with increased risk of complications such as pelvic inflammatory disease (PID) [4–6]. Repeat testing for chlamydia could identify chlamydia infections at an early stage of infection, thereby possibly reducing the individual risk of complications, and might lead to reduced transmission at the population level [4–6]. Therefore, several countries have established STI care guidelines on repeat testing for heterosexuals [7–10].

Repeat testing guidelines differ between countries with regard to the groups that are targeted, and the advised timing of the repeat test. A mathematical modelling study estimated the risk of a repeat chlamydia infection to be highest 2–5 months after treatment, suggesting a possible window for repeat testing in case of an initial positive test [11]. This is in line with the recommended test interval in most countries for repeat testing after chlamydia infection: the US centre for disease control and prevention recommends men and women to return preferably 3 months after treatment, and at least within 12 months [7]. In Australia, repeat testing at 3 months is recommended [8], and European and UK guidelines recommend repeat testing 3–6 months after treatment for both men and women aged <25 years [9, 10]. Repeat testing after a negative chlamydia test is not explicitly recommended, but annual chlamydia screening is advised in the European and US guidelines for all sexually active men and women aged <25, or women aged <25, respectively [7, 9].

In the Netherlands, guidelines on repeat testing are limited. For men who have sex with men, advice is given to test for STI every 6 or 3 months, depending on risk behaviour [12]. For heterosexuals, guidelines on repeat testing are only established for initial chlamydia positive persons, and differ between testing facilities. In general practitioner guidelines, it is advised to retest patients within 12 months after treatment of an initial infection [13]. STI clinic guidelines recommend repeat testing 4–6 months after treatment [12]. However, while the repeat testing behaviour of MSM has been studied in the past [14, 15], it is not well known to what extend the guidelines for heterosexuals are currently being adhered to. In addition, knowledge on repeat testing among heterosexual STI clinic visitors with an initial negative chlamydia test is limited. Besides initial chlamydia positive attendees, the initial negative attendees are also of interest because Dutch STI clinics are restricted to people at increased risk of STI. Initial negative visitors could therefore still be a potential target group for repeat testing.

Two Dutch studies assessed the uptake of repeat testing after chlamydia infection at STI clinics, with differing results. One study found that 33.4% of all chlamydia positive individuals returned for a repeat test 3–12 months after treatment [16], whereas the other study found 9.2% repeat testing at 5–8 months after treatment among young heterosexuals [17]. However, these studies used regional STI clinic data only, and did not assess repeat testing behaviour and the chlamydia prevalence at repeat consultation among visitors with an initial chlamydia negative test result.

To inform further specification of the Dutch guidelines, and to be able to evaluate them properly in the future, more insight is needed into current chlamydia repeat testing behaviour among Dutch heterosexual STI clinic visitors. In this study, we aimed to describe current repeat testing behaviour and to identify groups for which repeat testing advice could be advantageous. We do this by determining repeat testing uptake and by identifying predictors of repeat testing and predictors of chlamydia positivity at repeat test among heterosexual STI clinic visitors with both an initial negative or an initial positive chlamydia test.

## Methods

### Study design

This study used Dutch STI clinic surveillance data, containing all consultations from all STI outpatient clinics in the Netherlands. The STI clinic surveillance data contains information on personal characteristics, sexual behaviour, and STI diagnoses of each patient at each consultation. As STI testing at Dutch STI clinics is publicly funded, it is restricted to certain target groups who are considered at high risk for STI based on positivity rates obtained from the STI clinic surveillance data (further referred to as high risk populations) [18]. Heterosexual men and women aged 24 years or younger and MSM are considered at high risk for STI regardless behavioural or other risk factors. Heterosexual men and women aged 25 years and older are allowed to test at the clinics if they report at least one of the following factors: having STI symptoms, being notified by an infected partner, originating from or having a partner from a non-Western area, being a commercial sex worker, having had an STI in the past year, being a victim of sexual violence, or for women having an MSM partner [18].

For this study, we used data from June 1st 2014 to December 31st 2015, as in June 2014 a personal identification number was added to data collection, which enabled linkage of consultations performed on the same individuals at the same STI clinic. We selected consultations of heterosexual men and women who had at least one chlamydia test between June 2014 and December 2015. Individuals who reported having had sex with only the opposite gender in the past 6 months were defined as heterosexuals in this study. Thereby this term does not necessarily reflect the sexual identity of the individual, but rather their self-reported behaviour. Consultations where no chlamydia test was recorded, or that had missing information on chlamydia diagnosis were excluded. STI tests that were not performed at STI clinics, but at other health care settings such as general practitioners or hospitals could not be included, as systematically collected data are not available for these facilities.

The main outcome of interest was repeat testing, which was defined as returning to the STI clinic for a second consultation within 12 months after the initial visit. Individuals were defined as single testers if they had only one STI clinic visit during the study period, or if they returned to the STI clinic after more than 12 months from the initial visit. Although a test of cure (TOC) is generally not recommended in the Netherlands, consultations that were performed within 35 days after the initial consultation were deleted to minimise the risk that possible TOCs were included. Also, the 35 day cut off limits the chance of a repeat test being positive due to residual bacterial material from an already treated chlamydia infection at the initial consultation [19]. Persons with their first consultation within 35 days of the end of the study period (December 31st 2015) were excluded, as they had no opportunity for a repeat test. All analyses were performed using the first two consultations of each individual. Chlamydia positivity was defined as a diagnosed chlamydia infection at any location (urogenital, anorectal, or oral). Chlamydia diagnoses were based on nucleic acid amplification test (NAAT).

### Statistical analyses

All analyses were performed for men and women separately because of differences in chlamydia positivity rates and STI clinic visiting behaviour between women and men [18]. First, descriptive analyses were performed. We calculated the time between consecutive consultations of each individual, stratified by the initial test result. We also calculated chlamydia positivity of repeat tests, stratified by time between initial test and repeat test.

Second, we identified predictors of repeat testing using logistic regression analysis, comparing single testers with repeat testers. For this analysis, characteristics from the initial consultation were used, because the initial consultation is the moment when repeat testing advice based on person characteristics can be given. Age was included in three categories; 13–19, 20–24, and 25+, as in the Netherlands persons aged <20 have highest chlamydia positivity rates at STI clinics, and for persons aged >24 different triaging guidelines are in place [18]. Ethnicity was based on country of birth of both the participant and its parents, according to the definitions of Statistics Netherlands [20]. Persons from Western Europe, Northern America, and Australia were considered of Western non-Dutch ethnicity. All other ethnicities were grouped as non-Western, which is the same categorisation as used in triage at the STI clinics. An uncertainty analysis was performed to evaluate whether categorisation of ethnicity into more subgroups would provide different results. All other characteristics were self-reported, except for the chlamydia diagnoses. Some variables could not be included in the logistic regression due to small group sizes, and were only included in descriptive analyses (region of the STI clinic, being HIV positive, being (client of) commercial sex worker, and being a swinger).

Variables were included in multivariable analysis when significant in univariable analysis (*P* < 0.1). If a variable had more than 5% missing values, missings were included in the analysis as a separate category to reduce loss of data. A complete case analysis was performed as uncertainty analysis to evaluate robustness of the model for missing data. The multivariable model was made using backward elimination, using a significance level of 0.05. Because a chlamydia infection at the initial consultation could influence the identified predictors of repeat testing (these people are more likely to receive repeat testing advice), effect modification by chlamydia infection at initial consultation was assessed by adding interaction terms and stratifying analyses. Furthermore, since only 1.5 years of data was available, some people might have had limited opportunity to return for a repeat test within the timeframe of this study. To identify the consequences of this, uncertainty analyses were performed in which only persons with a minimum of 12 months of follow-up time were included.

Last, to assess predictors of having a chlamydia positive repeat test within 1 year after initial consultation, logistic regression analysis was done among the groups of repeat testers. Again, characteristics from the initial consultation and the same methods were used. However, missings were in this model not included as a separate category, as the number of individuals in these groups would become too small for multivariable analysis. The same variables were used as in the previous analysis, with the addition of time between first and repeat test, categorised in ≤6 months and >6 months. An uncertainty analysis was performed using characteristics from the consultation of the repeat test, instead of characteristics from the initial consultation. All analyses were performed using Stata v. 14 (StataCorp, College Station, TX).

## Results

### Study population

Between June 2014 and December 2015, 152,358 consultations were carried out at the STI clinics in the Netherlands among 127,650 heterosexual men and women. We excluded 324 consultations because there was missing chlamydia data or no chlamydia test was registered, and 563 consultations because they took place within 35 days of the previous consultation. Five thousand six hundred sixty-five persons were excluded because they did not have more than 35 days of follow-up time. Last, 5320 consultations were excluded because they were performed after the second consultation, leaving 140,486 consultations in 75,487 women and 46,286 men available for analysis. Characteristics at initial consultation stratified for women and men are shown in Additional file 1: Table S1.

### Timing and predictors of repeat testing

In total, 15.4% of women and 11.1% of men returned for at least one test within 1 year after the initial consultation (repeat testers), an overall repeat testing rate of 13.7% (Table 1). For STI clinic visitors with an initial chlamydia positive test, 21.1% returned for a repeat test and among visitors with an initial negative test, 12.5% returned for a repeat test. In the uncertainty analysis including only persons with a minimum of 12 months of follow-up time, the overall repeat testing increased to 22.7% among women and 16.7% among men. The median time between first test and repeat test was similar for women (171 days) and men (170 days). Both women and men with a positive initial test returned to the STI clinic for testing sooner compared to individuals with a negative initial test (Additional file 2: Figure S1).

**Table 1:** Testing characteristics of heterosexuals at Dutch STI clinics between June 2014 and December 2015

	Women	Men
Total number of consultations	88,409	52,077
Individuals with N consultations (%)		
1	62,565 (82.9)	40,495 (87.5)
2	12,922 (17.1)	5791 (12.5)
Total number of individuals	75,487	46,286
Individuals with a repeat test within 12 months (overall) (%)	11,612 (15.4)	5157 (11.1)
Repeat testers among initial CT negatives (%)	9136/64,842 (14.1)	3910/39,283 (10.0)
Repeat testers among initial CT positives (%)	2476/10,645 (23.3)	1247/7003 (17.8)
Median time between initial test and repeat test (overall), days (IQR)	171 (111–243)	170 (106–245)
After initial negative test	178 (116–249)	176 (112–252)
After initial positive test	147 (90–218)	147 (90–221)

*IQR* interquartile range, *CT* Chlamydia

The strongest predictors of repeat testing for men and women were having had more than four sex partners in the past 6 months (adjusted odds ratio [aOR] 2.27 for women and aOR 2.80 for men), and a chlamydia infection at initial consultation (aOR 2.00 for women and aOR 1.85 for men) (Tables 2 and 3). Furthermore, for women, variables predictive for repeat testing were Non-Western ethnicity, use of a condom at last sexual contact, reporting STI symptoms, and having had an STI in the past year. A slightly lower odds of repeat testing was observed for having a high education level, being of western non-Dutch ethnicity, and having received partner notification. Among men, results were comparable except for condom use and partner notification at the initial consultation, which were not associated with repeat testing. In addition, among women, age was not a predictor of repeat testing, but men aged 25 years and older were less likely to be repeat testers compared to 13 to 19 year olds.

**Table 2:** Predictors of repeat testing among heterosexual women at initial consultation, June 2014 to December 2015

	Single testers		Repeat testers					
					Crude		Adjusted	
	n	%	n	%	OR	95% CI	OR	95% CI
Total	63,875	84.6	11,612	15.4				
Age								
13–19	8543	13.4	1559	13.4	1	–	–	–
20–24	36,471	57.1	6623	57.0	0.99	(0.94–1.05)	–	–
25+	18,861	29.5	3430	29.5	1.00	(0.93–1.06)	–	–
Education level^a^								
Low/medium	17,554	27.5	3201	27.6	1	–	1	–
High	37,118	58.1	5422	46.7	0.80	(0.76–0.84)	0.88	(0.84–0.93)
Ethnicity								
Dutch	47,441	74.4	7906	68.2	1	–	1	–
Western non-Dutch	3718	5.8	613	5.3	0.99	(0.91–1.08)	0.91	(0.83–1.00)
Non-Western	12,650	19.8	3081	26.6	1.46	(1.40–1.53)	1.31	(1.26–1.39)
Number of sex partners in past 6 months								
0–1	21,107	33.7	2461	22.3	1	–	1	–
2–3	29,046	46.4	5162	46.7	1.52	(1.45–1.60)	1.56	(1.48–1.64)
4+	12,471	19.9	3421	31.0	2.35	(2.22–2.49)	2.27	(2.14–2.41)
Condom use at last sexual contact								
No	48,663	78.5	8214	72.6	1	–	1	–
Yes	13,294	21.5	3097	27.4	1.38	(1.32–1.44)	1.10	(1.04–1.15)
Received partner notification								
No	53,816	84.7	9953	86.1	1	–	1	–
Yes	9749	15.3	1610	13.9	0.89	(0.84–0.95)	0.80	(0.75–0.85)
Reported STI symptoms								
No	41,179	64.9	7253	62.8	1	–	1	–
Yes	22,257	35.1	4287	37.2	1.09	(1.05–1.14)	1.08	(1.03–1.12)
History of STI (CT/GO/SY)^a,b^								
No	52,597	82.4	9215	79.4	1	–	1	–
Yes	5072	7.9	1672	14.4	1.88	(1.77–2.00)	1.86	(1.74–1.98)
Chlamydia infection								
No	55,706	87.2	9136	78.7	1	–	1	–
Yes	8169	12.8	2476	21.3	1.85	(1.76–1.94)	2.00	(1.89–2.11)

Abbreviations: *CT* chlamydia, *GO* gonorrhoea, *SY* syphilis

^a^Missing values included in the analysis as a separate category (ORs not shown)

^b^In 2014, history of STI was asked regarding the past 2 years. In 2015 this changed to the past year only

**Table 3:** Predictors of repeat testing among heterosexual men at initial consultation, June 2014 to December 2015

	Single testers		Repeat testers					
					Crude		Adjusted	
	n	%	n	%	OR	95% CI	OR	95% CI
Total	41,129	88.9	5157	11.1				
Age								
13–19	2596	6.3	299	5.8	1	–	1	–
20–24	19,139	46.5	2582	50.1	1.17	(1.03–1.33)	1.08	(0.94–1.23)
25+	19,394	47.2	2276	44.1	1.02	(0.90–1.16)	0.82	(0.72–0.93)
Education level^a,c^								
Low	12,971	31.5	1516	29.4	1	–	1	–
High	22,363	54.4	2376	46.1	0.91	(0.85–0.97)	0.98	(0.91–1.06)
Ethnicity								
Dutch	26,964	65.6	3006	58.3	1	–	1	–
Western non-Dutch	2524	6.1	322	6.3	1.14	(1.01–1.29)	1.06	(0.93–1.20)
Non-Western	11,602	28.2	1828	35.4	1.41	(1.33–1.50)	1.37	(1.28–1.46)
Number of sex partners in past 6 months								
0–1	8559	21.0	529	10.4	1	–	1	–
2–3	16,191	39.7	1783	34.9	1.78	(1.61–1.97)	1.75	(1.58–1.94)
4+	16,023	39.3	2801	54.8	2.83	(2.57–3.12)	2.80	(2.54–3.09)
Condom use at last sexual contact								
No	29,257	73.8	3720	74.5	1	–	–	–
Yes	10,376	26.2	1273	25.5	0.96	(0.90–1.03)	–	–
Received partner notification								
No	30,763	75.2	3917	76.2	1	–	–	–
Yes	10,155	24.8	1223	23.8	0.95	(0.88–1.01)	–	–
Reported STI symptoms								
No	26,968	65.9	3135	61.1	1	–	1	–
Yes	13,939	34.1	1995	38.9	1.23	(1.16–1.31)	1.10	(1.03–1.17)
History of STI (CT/GO/SY)^a,b^								
No	34,088	82.9	4103	79.6	1	–	1	–
Yes	2610	6.4	721	14.0	2.30	(2.10–2.51)	2.08	(1.90–2.28)
Chlamydia infection								
No	35,373	86.0	3910	75.8	1	–	1	–
Yes	5756	14.0	1247	24.2	1.96	(1.83–2.10)	1.85	(1.72–1.99)

Abbreviations: *CT* chlamydia, *GO* gonorrhoea, *SY* syphilis

^a^Missing values included in the analysis as a separate category (ORs not shown)

^b^In 2014, history of STI was asked regarding the past 2 years. In 2015 this changed to the past year only

^c^Included in the multivariable model due to significance of the ‘missing values’ category (not shown)

Chlamydia infection at initial consultation was an effect modifier for repeat testing among both women and men. However, none of the directions of effect changed when analyses were stratified (Additional file 3: Table S2). Among both women and men, the effect of number of sex partners and history of STI was slightly weaker among initial chlamydia positives. Among men, the effect of age became stronger among initial chlamydia positives. The uncertainty analysis including persons with a minimum of 12 months of follow-up time, showed that predictors of repeat testing in the logistic regression analysis hardly changed. Only among women, the analysis showed that older women (>25) were less often repeat testers (Additional file 4: Table S3). Uncertainty analyses using complete cases only showed very little differences in the found predictors of repeat testing (compared to the main analyses), indicating that the analyses were also robust to missing values (Additional file 5: Table S4). Analyses including more subgroups for ethnicity did not change the results, and did not reveal a specific non-Western ethnicity group as predictor of repeat testing among women. Among men, Suriname, the Dutch Antilles and Sub-Saharan Africa were associated with more repeat testing in univariable analysis, but numbers in the different subgroups were too low to perform multivariable analysis (Additional file 6: Table S5).

### Repeat test positivity and predictors of positive repeat tests

**Fig. 1:**
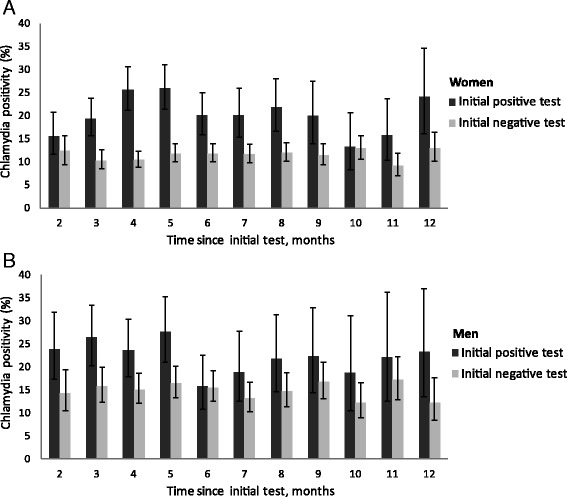
Chlamydia positivity at repeat test by month after initial test, split by initial test result in heterosexual women (**a**) and men (**b**) testing at STI outpatient clinics in the Netherlands between June 2014 and December 2015. Months were defined as 30 days, and tests performed within 35 days after the initial consultation were not included

At repeat test, 13.4% of women and 16.7% of men tested positive for chlamydia (compared to 14.1% and 15.1% at initial test). Both for women (Fig. 1a) and men (Fig. 1b), chlamydia positivity at repeat test was higher among individuals with a positive initial test compared to individuals with an initial negative test. Especially between 3 and 5 months after the initial test a clear difference can be seen between people with an initial positive and negative test, as positivity rates of over 25% were found in repeat tests after an initial positive test, while chlamydia positivity of repeat tests after an initial negative test remained constant over time.

Among the repeat testers, being aged >25 years greatly reduced the odds of a chlamydia positive repeat test among both women and men (aOR 0.44 and aOR 0.50, respectively) (Table 4). Furthermore, among women, a chlamydia infection at initial consultation was associated with higher risk for repeat infection, whereas condom use at last sexual contact showed a protective effect. Among men, significant interaction was found between reporting STI symptoms and chlamydia diagnosis; hence, a combined variable was made. For men, having both a chlamydia infection and reporting STI symptoms at initial consultation was associated with increased risk of repeat test positivity (aOR 2.30). Table 5 shows a side by side comparison of predictors of repeat testing and repeat test positivity.

**Table 4:** Predictors of having a chlamydia positive repeat test among repeat testers, using initial STI clinic consultation characteristics^a^

	Crude		Adjusted	
	OR	95% CI	OR	95% CI
Women				
Age				
13–19	1	–	1	–
20–24	0.77	(0.66–0.88)	0.82	(0.70–0.95)
25+	0.38	(0.32–0.45)	0.44	(0.37–0.53)
Condom use at last sexual contact				
No	1	–	1	–
Yes	0.69	(0.60–0.78)	0.83	(0.72–0.95)
Chlamydia infection at initial consultation				
No	1	–	1	–
Yes	2.04	(1.81–2.29)	1.83	(1.62–2.06)
Men				
Age				
13–19	1	–	1	–
20–24	0.77	(0.58–1.02)	0.82	(0.61–1.09)
25+	0.47	(0.35–0.63)	0.50	(0.37–0.66)
Combined variable, reported symptoms and chlamydia infection				
CT negative, no STI symptoms	1	–	1	–
CT negative, reported STI symptoms	1.06	(0.88–1.27)	1.12	(0.93–1.34)
CT positive, no STI symptoms	1.07	(0.84–1.37)	1.00	(0.79–1.28)
CT positive, reported STI symptoms	2.31	(1.89–2.83)	2.30	(1.88–2.82)

^a^Table only includes variables that remained significant in multivariate analysis

**Table 5:** Comparison of predictors of repeat testing and predictors of having a chlamydia positive repeat test

Characteristics	Associated with repeat testing	Associated with positive repeat test
Chlamydia infection	Yes	Yes
Young age	No	Yes
Non-Western ethnicity	Yes	No
More sex partners	Yes	No

The uncertainty analyses using characteristics from the consultation of the repeat test instead of the initial consultation showed similar results, but receiving partner notification and reporting symptoms also remained in the model, and became the strongest determinants for a positive repeat test (Additional file 7: Table S6). Uncertainty analyses for predictors of having a positive repeat test with persons with a minimum of 12 months follow-up time were not possible due to small sample size.

## Discussion

Overall, 15% of heterosexual women and 11% of heterosexual men visiting Dutch STI clinics between June 2014 and December 2015 returned to the same clinic for repeat testing within the study period. The strongest predictors of repeat testing were having had more than four sex partners in the past 6 months and having a chlamydia infection at the initial consultation. Reinfection rates were highest among initial chlamydia positives at three to 5 months after the initial consultation, with positivity rates over 25%. Chlamydia infection at the initial consultation and young age were strong predictors for having a chlamydia positive repeat test.

This study uses national STI clinic surveillance data, which results in a very large study population, allowing extensive analyses among both initial negative and initial positive visitors. However, there are also limitations to this study. First, linkage of consultations by ID number was only possible for individuals returning to the same STI clinic. Hence, (repeat) tests at other STI clinics or other testing facilities, such as general practitioners or online STI test providers, are not included and this might have resulted in an underestimation of the repeat testing rates. From 2016 onwards, STI clinics ask their attendees if and where they tested for STI in the past year. These new data might provide future insight in repeat testing between STI clinics and from other testing facilities to the STI clinics. Second, as this study used STI clinic surveillance data, only information on individuals who actively returned to the STI clinic was available. The positivity rates in people not returning to the clinics are unknown, which hampers generalisability of our results to all STI clinic visitors. Third, longitudinal data has only been available since June 2014, which resulted in a relative short follow-up time of 1.5 years. Consequently, we have underestimated the percentage of repeat testers as single testers might have returned for a repeat test after the follow-up period, and single testers might have been misclassified if the initial consultation in our data was actually already a repeat test. Uncertainty analyses showed that including only people with a minimum of 12 months follow-up did increase the percentage of repeat testers, but hardly changed the found determinants of repeat testing, indicating that our results were robust for follow-up time. Last, we only included the first two consultations of each individual. As only a small percentage of people had more than two consultations, we expect this exclusion not to have influenced our results.

We found that overall, 13.7% of heterosexuals visiting STI clinics returned for a repeat chlamydia test during our study period, and 20.5% when including only people with a minimum of 1 year follow-up. This is slightly lower than a study among UK genitourinary medicine clinic visitors, where a repeat chlamydia testing incidence of 26 per 100 person years was found [21]. Our study showed that 21.1% returned after an initial positive test, which was also lower than a study using US laboratory data, where repeat testing was 38% in women and 22% in men after an initial positive test [22]. Our estimate is, however, in the middle of two other regional estimates from the Netherlands (9.2% [17] and 33.4% [16] of repeat tests after an initial positive test). Differences between our study findings and others can be explained by differences in study population, definition of repeat test, and duration of follow-up. Among women, the percentage of repeat testers was higher than among men. This is in line with other Dutch studies on repeat testing [16, 17, 23]. This could be due to the fact that women are known to generally seek health-related information and health care more often than men [24, 25]. Last, we showed that repeat test positivity rates were highest among initial chlamydia positives between 3 and 5 months after the initial consultation, which is in line with findings from other studies [11, 26] and current repeat testing guidelines after CT infection elsewhere [7–10].

Predictors of repeat testing found in our study were mostly characteristics associated with- or indicative of sexual risk behaviour and risk of STI: higher number of partners, non-Western ethnicity [18, 27], history of STI infection, and chlamydia infection at initial consultation. This might indicate that individuals who are at increased risk are also more motivated to test repeatedly. However, it could also be a consequence of the STI clinic policies, since non-Western ethnicity is a triaging criterion and therefore facilitates admittance to the STI clinic. Strikingly, we did not find young age (<25) to be predictive of repeat testing among women in our main analysis, and only a small effect among men, despite the fact that being aged <25 also is an important triaging criterion. In the uncertainty analyses with persons with a minimum of 12 months follow-up time, women aged >25 were less often repeat testers, which was more in line with the triaging criteria. However, sample sizes were much smaller in the uncertainty analysis, making these results less reliable. Future research using longer time periods is warranted to determine these associations with more certainty.

The strongest predictor for a chlamydia positive repeat test, among repeat testers, was young age. This is in agreement with studies showing that young persons are at increased risk of chlamydia infection [18, 28, 29]. Initial chlamydia infection (women) and initial chlamydia infection combined with symptoms (men) were also strong predictors for having a positive repeat test. Chlamydia infection being a predictor for repeat infection is in line with findings from studies from the UK, US, and Sweden [21, 22, 30]. Our analysis was corrected for several risk behaviour parameters at initial consultation, such as number of partners, but the association between initial and repeat infection might be due to other behavioural or biological factors as well. For example, repeat infections could be a consequence of inadequate partner notification and/or treatment of the partner of the infected index case thereby increasing the probability of reinfection of the index case [31, 32]. Furthermore, treatment failure could also contribute to the association between initial and repeat positive test. In the Netherlands, people tested positive at the urogenital site receive 1 g of azithromycin, whereas those tested positive at the anorectal site receive 7-days (100 mg twice daily) doxycycline [33]. However, treatment failure after urogenital or anorectal infections for chlamydia is possible [34, 35]. For heterosexual men, chlamydia infection was only associated with repeat test positivity if symptoms were also reported. This might indicate that for women and men different mechanisms behind repeat infections are involved. A possible explanation might be that women can be reinfected from the anorectal site to the urogenital site through autoinoculation [36]. At the STI clinics, women are only tested for anorectal infection on indication, which is likely to miss anal infections in women who did not report recent anal intercourse [37]. When women are infected at both anatomic locations, but not tested at the anorectal site, they receive azithromycin, which showed reduced effectiveness for treating anorectal chlamydia infections [35]. This may cause higher rates of repeat infections due to autoinoculation in women, whereas for heterosexual men, autoinoculation is less likely [38].

When comparing the most important predictors of repeat testing with predictors of having a chlamydia positive repeat test among repeat testers, several important findings emerge. First, initial chlamydia infection was associated with both repeat testing and with repeat test positivity. This is an indication that initial positives are indeed at increased risk of repeat infection. It also shows that chlamydia positives are already returning to the STI clinic more often compared to initial chlamydia negatives, following guidelines. However, still only 21% of initial positives were repeat tester. Second, young age (<25) was not associated with repeat testing in the main analysis, and was a strong predictor of a chlamydia infection at repeat test. This indicates that young people may not be returning to the STI clinic more often, even though they are at the highest risk of infection at repeat test compared to the other ages. Therefore, young heterosexual STI clinic visitors could be an important target group for enhancing repeat testing. Third, non-Western ethnicity was associated with repeat testing. However, at repeat test, no difference in chlamydia positivity was seen between persons with non-Western ethnicity compared to persons with Dutch ethnicity. This finding was unexpected, as more STIs are generally found among those with non-Western ethnicity [18, 27]. This might indicate that the selection of persons with non-Western ethnicity that do return to the STI clinics for a repeat test, are not necessarily the ones that are at highest risk of obtaining STIs. Or, vice versa, that those with a Dutch ethnicity returning for a repeated test are the ones with increased risk of STI. The same thing was seen for number of partners; having more partners was associated with repeat testing, but not with positivity at the repeated test. This could also indicate that those returning are not necessarily the ones at highest risk.

## Conclusions

Altogether, the results of this study provide an extensive overview of current STI repeat testing behaviour among Dutch heterosexual STI clinic attendees, which can be used as guidance to determine for which groups repeat testing could be most advantageous. Our findings suggest that repeat testing could especially be beneficial for young persons (<25 years) with an initial chlamydia infection. However, interpretation of the results remains challenging due to the use of surveillance data, and since the motivations of people to return (or not return) to the clinic remain unknown. Furthermore, there is little insight into the current practices of STI clinics regarding whether (because of long waiting lists and high workload) and to whom repeat testing advice is given. Gaining insight into current practices is important to identify barriers and attitudes toward repeat testing among health care professionals, which should be taken into account when evaluating the guidelines. More research is needed to determine repeat testing strategies in the Netherlands in terms of (cost) effectiveness, feasibility, and public health impact.

## Additional files

Additional file 1: Table S1. Initial consultation characteristics among heterosexuals visiting Dutch STI clinics between June 2014 and December 2015. (DOCX 19 kb)

Additional file 2: Figure S1. The percentage of repeat tests by month, split by initial chlamydia test result in heterosexual women (A) and men (B) testing at STI outpatient clinics in the Netherlands between June 2014 and December 2015. Months were defined as 30 days and tests performed after 12 months or within 35 days of the initial consultation were not included. (EPS 998 kb)

Additional file 3: Table S2. Predictors of repeat testing among heterosexual women at initial STI clinic consultation between June 2014 and December 2015, stratified by chlamydia test result at initial consultation. Predictors of repeat testing among heterosexual men at initial STI clinic consultation between June 2014 and December 2015, stratified by chlamydia test result at initial consultation. (DOCX 19 kb)

Additional file 4: Table S3. Predictors of repeated testing at initial STI clinic consultation among heterosexual women and men with at least 12 months of follow-up time between June 2014 and December 2015. (DOCX 19 kb)

Additional file 5: Table S4. Predictors of repeat testing among heterosexual women and men at initial STI clinic consultation between June 2014 and December 2015: results from the original analyses versus complete case analysis. (DOCX 20 kb)

Additional file 6: Table S5. Predictors of repeat testing among heterosexual women and men at initial STI clinic consultation between June 2014 and December 2015, including multiple ethnicity subgroups. (DOCX 20 kb)

Additional file 7: Table S6. Determinants of having a chlamydia positive repeat test, using characteristics from repeat test. (DOCX 14 kb)

## Abbreviations

(a)OR: (adjusted) odds ratio
CI: Confidence interval
CT: Chlamydia trachomatis
GO: Gonorrhoea
IQR: Interquartile range
MSM: Men who have sex with men
PID: Pelvic inflammatory disease
STI: Sexually transmitted infection
SY: Syphilis
TOC: Test of cure
