# Supplementary material for: Repeat Chlamydia trachomatis testing among heterosexual STI outpatient clinic visitors in the Netherlands: a longitudinal study
Source: BMC Infect Dis. 2017 Dec 20;17:782. doi: 10.1186/s12879-017-2871-1 (PMC5738891; doi:10.1186/s12879-017-2871-1)
Supplement: Supplementary file 1 — Initial consultation characteristics among heterosexuals visiting Dutch STI clinics between June 2014 and December 2015. (DOCX 19 kb) [file 12879_2017_2871_MOESM1_ESM.docx]

| **Table** Initial consultation characteristics among heterosexuals at Dutch STI clinics between June 2014 and December 2015 | | | | |
| --- | --- | --- | --- | --- |
|  | **Women** | | **Men** | |
|  | **n** | **%** | **n** | **%** |
| **Total** | 75,487 |  | 46,286 |  |
| **Age** |  |  |  |  |
| 13-19 | 10,102 | 13.4 | 2,895 | 6.3 |
| 20-24 | 43,094 | 57.1 | 21,721 | 46.9 |
| 25+ | 22,291 | 29.5 | 21,670 | 46.8 |
| **Education level** |  |  |  |  |
| Low | 20,755 | 27.5 | 14,488 | 31.3 |
| High | 42,540 | 56.3 | 24,739 | 53.4 |
| Missing values | 12,192 | 16.2 | 7,059 | 15.3 |
| **Ethnicity** |  |  |  |  |
| Dutch | 55,347 | 73.3 | 29,970 | 64.7 |
| Western non-Dutch | 4,331 | 5.7 | 2,846 | 6.2 |
| Non-Western | 15,731 | 20.8 | 13,430 | 29.0 |
| Missing values | 78 | 0.1 | 40 | 0.1 |
| **Region of STI clinic** |  |  |  |  |
| Limburg | 4,834 | 6.4 | 3,270 | 7.1 |
| Noord-Holland/Flevoland | 29,564 | 39.2 | 17,081 | 36.9 |
| Noord-Nederland | 4,875 | 6.5 | 2,141 | 4.6 |
| Oost-Nederland | 10,051 | 13.3 | 6,313 | 13.6 |
| Utrecht | 3,799 | 5.0 | 1,884 | 4.1 |
| Zeeland/Brabant | 9,223 | 12.2 | 5,914 | 12.8 |
| Zuid-Holland Noord | 3,948 | 5.2 | 2,896 | 6.3 |
| Zuid-Holland Zuid | 9,193 | 12.2 | 6,787 | 14.7 |
| **Number of sex partners in past 6 months** |  |  |  |  |
| 0-1 | 23,568 | 31.2 | 9,088 | 19.6 |
| 2-3 | 34,208 | 45.3 | 17,974 | 38.8 |
| 4+ | 15,892 | 21.1 | 18,824 | 40.7 |
| Missing values | 1,819 | 2.4 | 400 | 0.8 |
| **Condom use at last sexual contact** |  |  |  |  |
| No | 56,877 | 75.4 | 32,977 | 71.2 |
| Yes | 16,391 | 21.7 | 11,649 | 25.2 |
| Missing values | 2,219 | 2.9 | 1,660 | 3.6 |
| **Received partner notification** |  |  |  |  |
| No | 63,769 | 84.5 | 34,680 | 74.9 |
| Yes | 11,359 | 15.0 | 11,378 | 24.6 |
| Missing values | 359 | 0.5 | 228 | 0.5 |
| **Reported STI symptoms** |  |  |  |  |
| No | 48,432 | 64.2 | 30,103 | 65.0 |
| Yes | 26,544 | 35.1 | 15,934 | 34.4 |
| Missing values | 511 | 0.7 | 249 | 0.6 |
| **HIV positive** |  |  |  |  |
| No | 73,709 | 97.6 | 45,258 | 97.8 |
| Yes | 40 | 0.1 | 35 | 0.1 |
| Missing values | 1,738 | 2.3 | 993 | 2.1 |
| **History of STI (CT/GO/SY)†** |  |  |  |  |
| No | 61,812 | 81.9 | 38,191 | 82.5 |
| Yes | 6,744 | 8.9 | 3,331 | 7.2 |
| Missing values | 6,931 | 9.2 | 4,764 | 10.3 |
| **Commercial sex worker** |  |  |  |  |
| No | 70,723 | 93.7 | 45,638 | 98.6 |
| Yes | 4,170 | 5.5 | 226 | 0.5 |
| Missing values | 594 | 0.8 | 422 | 0.9 |
| **Client of commercial sex worker** |  |  |  |  |
| No | 74,591 | 98.8 | 41,822 | 90.4 |
| Yes | 211 | 0.3 | 4,088 | 8.8 |
| Missing values | 685 | 0.9 | 376 | 0.8 |
| **Swingers** |  |  |  |  |
| No | 12,494 | 16.6 | 7,932 | 17.1 |
| Yes | 551 | 0.7 | 777 | 1.7 |
| Missing values | 62,442 | 82.7 | 37,577 | 81.2 |
| **Chlamydia diagnosis** |  |  |  |  |
| Negative | 64,842 | 85.9 | 39,283 | 84,9 |
| Urogenital | 7,741 | 10.3 | 6,933 | 15,0 |
| Urogenital + other (anorectal/oral) | 2,345 | 3.11 | 18 | 0 |
| Other (anorectal/oral) | 559 | 0.7 | 52 | 0,1 |
| Abbreviations: CT chlamydia GO gonorrhoea SY syphilis  † In 2014, history of STI was asked regarding the past 2 years. In 2015 this changed to the past year only. | | | | |
