# Supplementary material for: Repeat Chlamydia trachomatis testing among heterosexual STI outpatient clinic visitors in the Netherlands: a longitudinal study
Source: BMC Infect Dis. 2017 Dec 20;17:782. doi: 10.1186/s12879-017-2871-1 (PMC5738891; doi:10.1186/s12879-017-2871-1)
Supplement: Supplementary file 3 — Predictors of repeat testing among heterosexual women at initial STI clinic consultation between June 2014 and December 2015, stratified by chlamydia test result at initial consultation. Predictors of repeat testing among heterosexual men at initial STI clinic consultation between June 2014 and December 2015, stratified by chlamydia test result at initial consultation. (DOCX 19 kb) [file 12879_2017_2871_MOESM3_ESM.docx]

| **Table** Predictors of repeat testing among heterosexual women at initial STI clinic consultation between June 2014 and December 2015, stratified by chlamydia test result at initial consultation | | | | |
| --- | --- | --- | --- | --- |
| **WOMEN** | **initial CT negative** | | **initial CT positive** | |
|  | **aOR** | **95% CI** | **aOR** | **95% CI** |
| **Education level*** |  |  |  |  |
| Low | 1 | - | 1 | - |
| High | **0.87** | **(0.82-0.92)** | 0.95 | (0.86-1.06) |
| **Ethnicity** |  |  |  |  |
| Dutch | 1 | - | 1 | - |
| Western non-Dutch | 0.91 | (0.82-1.01) | 0.91 | (0.73-1.13) |
| Non-Western | **1.33** | **(1.25-1.40)** | **1.28** | **(1.14-1.43)** |
| **Number of sex partners in past 6 months** |  |  |  |  |
| 0-1 | 1 | - | 1 | - |
| 2-3 | **1.62** | **(1.52-1.72)** | **1.32** | **(1.18-1.48)** |
| 4+ | **2.45** | **(2.29-2.62)** | **1.61** | **(1.40-1.83)** |
| **Condom use at last sexual contact** |  |  |  |  |
| No | 1 | - | 1 | - |
| Yes | **1.13** | **(1.07-1.19)** | 0.92 | (0.82-1.05) |
| **Received partner notification** |  |  |  |  |
| No | 1 | - | 1 | - |
| Yes | **0.85** | **(0.78-0.92)** | **0.69** | **(0.62-0.77)** |
| **Reported STI symptoms** |  |  |  |  |
| No | 1 | - | 1 | - |
| Yes | **1.06** | **(1.01-1.11)** | **1.15** | **(1.05-1.26)** |
| **History of STI (CT/GO/SY)*†** |  |  |  |  |
| No | 1 | - | 1 | - |
| Yes | **2.01** | **(1.87-2.16)** | **1.35** | **(1.17-1.56)** |
| Abbreviations: CT chlamydia GO gonorrhoea SY syphilis  * Missing values included in the analysis as a separate category (ORs not shown)  † In 2014, history of STI was asked regarding the past 2 years. In 2015 this changed to the past year only  Table only includes variables that remained significant in multivariate analysis | | | | |

| **Table** Predictors of repeat testing among heterosexual men at initial STI clinic consultation between June 2014 and December 2015, stratified by chlamydia test result at initial consultation | | | | |
| --- | --- | --- | --- | --- |
| **MEN** | **initial CT negative** | | **initial CT positive** | |
|  | **aOR** | **95% CI** | **aOR** | **95% CI** |
| **Age** |  |  |  |  |
| 13-19 | 1 | - | 1 | - |
| 20-24 | 1.17 | (1.00-1.37) | 0.89 | (0.70-1.12) |
| 25+ | 0.89 | (0.76-1.05) | **0.67** | **(0.53-0.85)** |
| **Education level*** |  |  |  |  |
| Low | 1 | - | 1 | - |
| High | 0.97 | (0.89-1.05) | 1.04 | (0.89-1.20) |
| **Ethnicity** |  |  |  |  |
| Dutch | 1 | - | 1 | - |
| Western non-Dutch | 1.05 | (0.92-1.21) | 1.06 | (0.80-1.41) |
| Non-Western | **1.38** | **(1.28-1.49)** | **1.31** | **(1.14-1.51)** |
| **Number of sex partners in past 6 months** |  |  |  |  |
| 0-1 | 1 | - | 1 | - |
| 2-3 | **1.88** | **(1.67-2.12)** | **1.40** | **(1.15-1.71)** |
| 4+ | **3.20** | **(2.87-3.60)** | **1.75** | **(1.44-2.13)** |
| **Reported STI symptoms** |  |  |  |  |
| No | 1 | - | 1 | - |
| Yes | **1.08** | **(1.00-1.16)** | **1.16** | **(1.02-1.32)** |
| **History of STI (CT/GO/SY)*†** |  |  |  |  |
| No | 1 | - | 1 | - |
| Yes | **2.14** | **(1.92-2.38)** | **1.91** | **(1.59-2.30)** |
| Abbreviations: CT chlamydia GO gonorrhoea SY syphilis  * Missing values included in the analysis as a separate category (ORs not shown)  † In 2014, history of STI was asked regarding the past 2 years. In 2015 this changed to the past year only.  Table only includes variables that remained significant in multivariate analysis | | | | |
