# Supplementary material for: Repeat Chlamydia trachomatis testing among heterosexual STI outpatient clinic visitors in the Netherlands: a longitudinal study
Source: BMC Infect Dis. 2017 Dec 20;17:782. doi: 10.1186/s12879-017-2871-1 (PMC5738891; doi:10.1186/s12879-017-2871-1)
Supplement: Supplementary file 4 — Predictors of repeated testing at initial STI clinic consultation among heterosexual women and men with at least 12 months of follow-up time between June 2014 and December 2015. (DOCX 19 kb) [file 12879_2017_2871_MOESM4_ESM.docx]

| **Table** Predictors of repeated testing at initial STI clinic consultation among heterosexual women and men with at least 12 months of follow-up time between June 2014 and December 2015. | | | | | | | | |
| --- | --- | --- | --- | --- | --- | --- | --- | --- |
|  | **women (n total=31,990)** | | | | **men (n total=19,207)** | | | |
|  | **n repeat testers** | **%** | **aOR** | **95% CI** | **n repeat testers** | **%** | **aOR** | **95% CI** |
| **Total** | 7,276 | 22.7 |  |  | 3,206 | 16.7 |  |  |
| **Age** |  |  |  |  |  |  |  |  |
| 13-19 | 996 | 23.7 | 1 | - | 188 | 16.1 | 1 | - |
| 20-24 | 4,065 | 23.0 | 0.91 | (0.83-1.01) | 1,510 | 18.5 | 1.08 | (0.90-1.30) |
| 25+ | 2,215 | 21.9 | **0.58** | **(0.52-0.65)** | 1,508 | 15.3 | **0.82** | **(0.68-0.98)** |
| **Education level** |  |  |  |  |  |  |  |  |
| Low | 1,712 | 23.3 | 1 | - | 802 | 15.9 | - | - |
| High | 2,925 | 19.8 | **0.87** | **(0.81-0.94)** | 1,217 | 14.2 | - | - |
| **Ethnicity** |  |  |  |  |  |  |  |  |
| Dutch | 4,938 | 20.9 | 1 | - | 1,893 | 14.8 | 1 | - |
| Western non-Dutch | 398 | 21.9 | 0.99 | (0.84-1.17) | 213 | 18.0 | **1.30** | **(1.10-1.53)** |
| Non-Western | 1,935 | 29.7 | **1.44** | **(1.32-1.58)** | 1,099 | 21.2 | **1.56** | **(1.42-1.70)** |
| **Number of sex partners in past 6 months** |  |  |  |  |  |  |  |  |
| 0-1 | 1,488 | 15.7 | 1 | - | 317 | 9.2 | 1 | - |
| 2-3 | 3,227 | 22.1 | **1.55** | **(1.43-1.69)** | 1,076 | 14.5 | **1.67** | **(1.45-1.92)** |
| 4+ | 2,196 | 31.4 | **2.46** | **(2.23-2.72)** | 1,778 | 21.7 | **2.89** | **(2.53-3.31)** |
| **Condom use at last sexual contact** |  |  |  |  |  |  |  |  |
| No | 5,159 | 21.5 | - | - | 2,310 | 17.0 | 1 | - |
| Yes | 1,991 | 27.0 | - | - | 815 | 15.9 | **0.89** | **(0.81-0.97)** |
| **Received partner notification** |  |  |  |  |  |  |  |  |
| No | 6,279 | 22.9 | 1 | - | 2,456 | 16.6 | - | - |
| Yes | 964 | 21.7 | **0.84** | **(0.76-0.94)** | 741 | 17.1 | - | - |
| **Reported STI symptoms** |  |  |  |  |  |  |  |  |
| No | 4,433 | 22.1 | 1 | - | 1,977 | 15.7 | 1 | - |
| Yes | 2,799 | 23.9 | **1.11** | **(1.03-1.19)** | 1,215 | 18.6 | **1.11** | **(1.02-1.21)** |
| **History of STI (CT/GO/SY)** |  |  |  |  |  |  |  |  |
| No | 5,662 | 21.7 | 1 | - | 2,549 | 16.1 | 1 | - |
| Yes | 1,284 | 35.0 | **1.82** | **(1.66-2.00)** | 532 | 28.7 | **1.85** | **(1.65-2.08)** |
| **Chlamydia infection** |  |  |  |  |  |  |  |  |
| No | 5,853 | 21.1 | 1 | - | 2,515 | 15.2 | 1 | - |
| Yes | 1,432 | 33.3 | **1.90** | **(1.73-2.09)** | 691 | 25.8 | **1.75** | **(1.58-1.95)** |
| Abbreviations: CT chlamydia GO gonorrhoea SY syphilis | | | | | | | | |
