# Supplementary material for: Repeat Chlamydia trachomatis testing among heterosexual STI outpatient clinic visitors in the Netherlands: a longitudinal study
Source: BMC Infect Dis. 2017 Dec 20;17:782. doi: 10.1186/s12879-017-2871-1 (PMC5738891; doi:10.1186/s12879-017-2871-1)
Supplement: Supplementary file 5 — Predictors of repeat testing among heterosexual women and men at initial STI clinic consultation between June 2014 and December 2015: results from the original analyses versus complete case analysis. (DOCX 20 kb) [file 12879_2017_2871_MOESM5_ESM.docx]

| **Table** Predictors of repeat testing among heterosexual women and men at initial STI clinic consultation between June 2014 and December 2015: results from the original analyses versus complete case analysis | | | | | | | | |
| --- | --- | --- | --- | --- | --- | --- | --- | --- |
|  | **Women** | | | | **Men** | | | |
|  | Original analysis | | Complete case analysis | | Original analysis | | Complete case analysis | |
|  | **aOR** | **95% CI** | **aOR** | **95% CI** | **aOR** | **95% CI** | **aOR** | **95% CI** |
| **Total** |  |  |  |  |  |  |  |  |
| **Age** |  |  |  |  |  |  |  |  |
| 13-19 | - | - | - | - | 1 | - | 1 | - |
| 20-24 | - | - | - | - | 1.08 | (0.94-1.23) | 1.05 | (0.90-1.21) |
| 25+ | **-** | **-** | - | - | **0.82** | **(0.72-0.93)** | **0.79** | **(0.68-0.93)** |
| **Education level** |  |  |  |  |  |  |  |  |
| Low/medium | 1 | - | 1 | - | 1 | - | 1 | - |
| High | **0.88** | **(0.84-0.93)** | **0.85** | **(0.81-0.90)** | 0.98 | (0.91-1.06) | 0.97 | (0.90-1.04) |
| missing | **1.73** | **(1.63-1.84)** | - | - | **2.01** | **(1.84-2.19)** | - | - |
| **Ethnicity** |  |  |  |  |  |  |  |  |
| Dutch | 1 | - | 1 | - | 1 | - | 1 | - |
| Western non-Dutch | 0.91 | (0.83-1.00) | 0.94 | (0.84-1.05) | 1.06 | (0.93-1.20) | 1.06 | (0.91-1.24) |
| Non-Western | **1.31** | **(1.26-1.39)** | **1.26** | **(1.19-1.34)** | **1.37** | **(1.28-1.46)** | **1.37** | **(1.26-1.48)** |
| **Number of sex partners in past 6 months** |  |  |  |  |  |  |  |  |
| 0-1 | 1 | - | 1 | - | 1 | - | 1 | - |
| 2-3 | **1.56** | **(1.48-1.64)** | **1.51** | **(1.42-1.60)** | **1.75** | **(1.58-1.94)** | **1.65** | **(1.46-1.86)** |
| 4+ | **2.27** | **(2.14-2.41)** | **2.13** | **(1.99-2.29)** | **2.80** | **(2.54-3.09)** | **2.64** | **(2.35-2.95)** |
| **Condom use at last sexual contact** |  |  |  |  |  |  |  |  |
| No | 1 | - | 1 | - | - | - | - | - |
| Yes | **1.10** | **(1.04-1.15)** | **1.08** | **(1.02-1.15)** | - | - | - | - |
| **Received partner notification** |  |  |  |  |  |  |  |  |
| No | 1 | - | 1 | - | - | - | - | - |
| Yes | **0.80** | **(0.75-0.85)** | **0.76** | **(0.70-0.81)** | - | - | - | - |
| **Reported STI symptoms** |  |  |  |  |  |  |  |  |
| No | 1 | - | 1 | - | 1 | - | 1 | - |
| Yes | **1.08** | **(1.03-1.12)** | **1.06** | **(1.01-1.12)** | **1.10** | **(1.03-1.17)** | **1.09** | **(1.02-1.18)** |
| **History of STI (CT/GO/SY)†** |  |  |  |  |  |  |  |  |
| No | 1 | - | 1 | - | 1 | - | 1 | - |
| Yes | **1.86** | **(1.74-1.98)** | **1.81** | **(1.68-1.94)** | **2.08** | **(1.90-2.28)** | **1.94** | **(1.74-2.15)** |
| missing | **0.73** | **(0.67-0.79)** | - | - | **0.70** | **(0.62-0.79)** | - | - |
| **Chlamydia infection** |  |  |  |  |  |  |  |  |
| No | 1 | - | 1 | - | 1 | - | 1 | - |
| Yes | **2.00** | **(1.89-2.11)** | **2.04** | **(1.68-1.94)** | **1.85** | **(1.72-1.99)** | **1.83** | **(1.69-2.00)** |

Abbreviations: CT chlamydia GO gonorrhoea SY syphilis

† In 2014, history of STI was asked regarding the past 2 years. In 2015 this changed to the past year only
