# Supplementary material for: Repeat Chlamydia trachomatis testing among heterosexual STI outpatient clinic visitors in the Netherlands: a longitudinal study
Source: BMC Infect Dis. 2017 Dec 20;17:782. doi: 10.1186/s12879-017-2871-1 (PMC5738891; doi:10.1186/s12879-017-2871-1)
Supplement: Supplementary file 6 — Predictors of repeat testing among heterosexual women and men at initial STI clinic consultation between June 2014 and December 2015, including multiple ethnicity subgroups. (DOCX 20 kb) [file 12879_2017_2871_MOESM6_ESM.docx]

| **Table** Predictors of repeat testing among heterosexual women and men at initial STI clinic consultation between June 2014 and December 2015, including multiple ethnicity subgroups. | | | | | | |
| --- | --- | --- | --- | --- | --- | --- |
|  | **WOMEN** | | | | **MEN** | |
|  | **Crude** | | **Adjusted** | | **Crude** | |
|  | **OR** | **95% CI** | **aOR** | **95% CI** | **OR** | **95% CI** |
| **Total** |  |  |  |  |  |  |
| **Age** |  |  |  |  |  |  |
| 13-19 |  |  | - | - |  |  |
| 20-24 |  |  |  |  |  |  |
| 25+ |  |  |  |  |  |  |
| **Education level*** |  |  |  |  |  |  |
| Low/medium |  |  | 1 | - |  |  |
| High |  |  | 0.88 | (0.84-0.93) |  |  |
| **Ethnicity** |  |  |  |  |  |  |
| Netherlands | 1 | - | 1 | - | 1 | - |
| Western Europe | 1.03 | (0.93-1.13) | 0.96 | (0.87-1.06) | 1.14 | (0.99-1.30) |
| North America / Australia | 0.81 | (0.64-1.01) | 0.72 | (0.58-0.91) | 1.18 | (0.86-1.59) |
| Turkey | 1.23 | (1.01-1.49) | 1.27 | (1.04-1.56) | 1.06 | (0.88-1.27) |
| Morocco / North Africa | 1.34 | (1.16-1.56) | 1.31 | (1.11-1.53) | 1.06 | (0.91-1.23) |
| Surinam | 1.49 | (1.37-1.63) | 1.49 | (1.36-1.63) | 1.90 | (1.72-2.09) |
| Dutch Antilles | 1.28 | (1.12-1.45) | 1.30 | (1.14-1.49) | 1.56 | (1.36-1.78) |
| Eastern Europe | 2.20 | (2.01-2.42) | 1.50 | (1.34-1.68) | 1.16 | (0.91-1.47) |
| Sub-Saharan Africa | 1.28 | (1.12-1.46) | 1.28 | (1.11-1.47) | 1.58 | (1.37-1.82) |
| Central and South America | 1.64 | (1.45-1.86) | 1.29 | (1.12-1.48) | 1.15 | (0.92-1.45) |
| Asia | 1.12 | (1.01-1.24) | 1.09 | (0.98-1.21) | 1.19 | (1.04-1.37) |
| **Number of sex partners in past 6 months** |  |  |  |  |  |  |
| 0-1 |  |  | 1 | - |  |  |
| 2-3 |  |  | 1.56 | (1.48-1.65) |  |  |
| 4+ |  |  | 2.27 | (2.14-2.41) |  |  |
| **Condom use at last sexual contact** |  |  |  |  |  |  |
| No |  |  | 1 | - |  |  |
| Yes |  |  | 1.09 | (1.04-1.15) |  |  |
| **Received partner notification** |  |  |  |  |  |  |
| No |  |  | 1 | - |  |  |
| Yes |  |  | 0.80 | (0.75-0.85) |  |  |
| **Reported STI symptoms** |  |  |  |  |  |  |
| No |  |  | 1 | - |  |  |
| Yes |  |  | 1.08 | (1.03-1.12) |  |  |
| **History of STI (CT/GO/SY)*†** |  |  |  |  |  |  |
| No |  |  | 1 | - |  |  |
| Yes |  |  | 1.86 | (1.74-1.98) |  |  |
| **Chlamydia infection** |  |  |  |  |  |  |
| No |  |  | 1 | - |  |  |
| Yes |  |  | 2.00 | (1.89-2.11) |  |  |
| Abbreviations: CT chlamydia GO gonorrhoea SY syphilis | | | | | | |
| * Missing values included in the analysis as a separate category (ORs not shown) | | | | | | |
| † In 2014, history of STI was asked regarding the past 2 years. In 2015 this changed to the past year only. | | | | | | |
