# Supplementary material for: Repeat Chlamydia trachomatis testing among heterosexual STI outpatient clinic visitors in the Netherlands: a longitudinal study
Source: BMC Infect Dis. 2017 Dec 20;17:782. doi: 10.1186/s12879-017-2871-1 (PMC5738891; doi:10.1186/s12879-017-2871-1)
Supplement: Supplementary file 7 — Determinants of having a chlamydia positive repeat test, using characteristics from repeat test. (DOCX 14 kb) [file 12879_2017_2871_MOESM7_ESM.docx]

| **Table** Determinants of having a chlamydia positive repeat test, using characteristics from repeat test. | | | | |
| --- | --- | --- | --- | --- |
|  | **WOMEN** | | **MEN** | |
|  | **aOR** | **95% CI** | **aOR** | **95% CI** |
| **Age** |  |  |  |  |
| 13-19 | 1 | - | 1 | - |
| 20-24 | **0.83** | **(0.70-0.99)** | 0.81 | (0.57-1.17) |
| 25+ | **0.40** | **(0.33-0.49)** | **0.41** | **(0.28-0.59)** |
| **Condom use at last sexual contact** |  |  |  |  |
| No | 1 | - |  |  |
| Yes | **0.77** | **(0.70-0.88)** |  |  |
| **Received partner notification** |  |  |  |  |
| No | 1 | - | 1 | - |
| yes | **3.10** | **(2.74-3.52)** | **2.45** | **(2.08-2.88)** |
| **Reported STI symptoms** |  |  |  |  |
| No | 1 | - | 1 | - |
| Yes | **1.34** | **(1.19-1.49)** | **2.61** | **(2.23-3.05)** |
| **Chlamydia infection at initial consultation** |  |  |  |  |
| No | 1 | - | 1 | - |
| Yes | **1.73** | **(1.53-1.96)** | **1.44** | **(1.22-1.69)** |
